# Clinical and radiological outcomes of dynamic navigation in endodontic microsurgery: a prospective study

**DOI:** 10.1007/s00784-023-05152-6

**Published:** 2023-08-02

**Authors:** Chen Chen, Rui Zhang, Wei Zhang, Fangzhe Li, Zan Wang, Li Qin, Yun Chen, Zhuan Bian, Liuyan Meng

**Affiliations:** 1 The State Key Laboratory of Oral & Maxillofacial Reconstruction and Regeneration, Key Laboratory of Oral Biomedicine Ministry of Education, Hubei Key Laboratory of Stomatology, School & Hospital of Stomatology, Wuhan University, Wuhan, China; 2 Department of Cariology and Endodontics, School and Hospital of Stomatology, Wuhan University, Wuhan, China; 3 Suzhou Digital-Health Care Co. Ltd, Suzhou, China

**Keywords:** Dynamic navigation, Endodontic microsurgery, Prognosis, Guided endodontics, Trephine

## Abstract

**Objectives:**

This study was aimed at evaluating the clinical and radiological outcomes of novel dynamic navigation (DN)–aided endodontic microsurgery (EMS), with an analysis of potential prognostic factors.

**Materials and methods:**

Forty-six teeth from 32 patients who received DN-aided EMS were included. Clinical and radiographic assessments were performed at least 1 year postoperatively. Two calibrated endodontists assessed radiological outcomes according to two-dimensional (2D) periapical radiography (PA) and three-dimensional (3D) cone-beam computed tomography (CBCT) imaging using Rud’s and Molven’s criteria and modified PENN 3D criteria, respectively. Fisher’s exact test was used for statistical analysis of the predisposing factors.

**Results:**

Of the 32 patients with 46 treated teeth, 28 with 40 teeth were available for follow-up. Of the 28 patients, four (five teeth) refused to undergo CBCT and only underwent clinical and PA examinations, and the remaining 24 (35 teeth) underwent clinical, PA, and CBCT examinations. Combined clinical and radiographic data revealed a 95% (38/40) success rate in 2D healing evaluations and a 94.3% (33/35) success rate in 3D healing evaluations. No significant effect was found in sex, age, tooth type, arch type, preoperative lesion volume, preoperative maximum lesion size, presence/absence of crown and post, and the root canal filling state on the outcome of DN-aided EMS.

**Conclusions:**

DN-aided EMS has a favorable prognosis and could be considered an effective and reliable treatment strategy. Further investigations with larger sample sizes are required to confirm these results.

**Clinical relevance:**

DN-aided EMS could be considered an effective and reliable treatment strategy.

## Introduction

As a predictable and reliable resort for treating persistent apical periodontitis, endodontic microsurgery (EMS) has achieved a high success rate of 94% based on evidence-based literature [1]. Resecting a 3-mm root end perpendicular to the long axis of the root could remove 98% of apical ramifications and is indicated for the principle of modern EMS [2]. The experience and perceptual skills of the operator are highly associated with the outcomes of freehand EMS. Operator-related risks, including excessive bone removal, incorrect root-end resection length and bevel, and injury to important anatomical structures, may occur, particularly in anatomically challenging scenarios. To address these problems and achieve predictable outcomes, guided EMS was introduced into the field of endodontics using novel computer-aided treatment methods. The guided methods include static navigation (SN) and dynamic navigation (DN). Several in vitro studies have shown high-accuracy results for SN and DN in guided osteotomy and root-end resection [3, 4]. Guided EMS replaces the carbide or diamond bur with a rotated trephine bur, which is conventionally used for the effective removal of implants and bone graft materials [5]. In guided EMS, the surgical path for osteotomy and root-end resection is designed and performed with sufficient consideration of the principles of modern surgery. SN-aided EMS is achieved using a three-dimensional (3D) printer for template fabrication with sleeves and a coordinated trephine bur. The DN-aided EMS provides real-time visualization and full guidance of the drill through various anatomic structures. The location, angulation, and depth of the surgical path can be optimally determined preoperatively. Compared with freehand EMS, guided EMS presents a more minimized atraumatic access and accurate root-end resection length and bevel.

Periapical radiography (PA) has traditionally been used to evaluate the radiological outcomes of EMS. The criteria established by Rud et al. (1972) and Molven et al. (1987) are used to assess the healing outcome of EMS [6, 7]. However, the overlap of hard and soft tissues with different radiodensities, existing background noise, and geometric distortion is a limitation of PA in detecting periapical lesions [8]. Cone-beam computed tomography (CBCT) acquires views by 3D projection that presents the target anatomical structure in the axial, coronal, and sagittal views. In the field of endodontics, CBCT has been used for endodontic diagnosis, morphological evaluation of the root canal, and preoperative planning in EMS [9]. CBCT can provide an accurate assessment of the location and size of periapical radiolucency and yield a more sensitive and specific result than PA [10, 11]. In 2017, Schloss et al. proposed a detailed evaluation grading of modified PENN 3D criteria for surgical outcome [12].

In the past decade, numerous studies have analyzed prognostic factors for EMS. Several studies have indicated that age [13, 14], sex [13–15], tooth location and type [15–18], lesion size [13, 14, 16], presence of crown and post [19], and root filling quality [13, 17] are potential prognostic factors for EMS. Modern EMS advocates a minimized operation for bone removal and precise root-end resection. Buniag et al. (2021) reported a retrospective study of targeted EMS with SN that yielded a 91.7% success rate [20]. However, the success rate and prognostic factors of DN-aided EMS with trephine have not yet been investigated. Therefore, this study was aimed at investigating the clinical and radiological outcomes of the novel DN-aided EMS. Radiological outcomes were evaluated using PA with Rud’s and Molven’s criteria and CBCT with modified PENN 3D criteria for at least 1 year of follow-up. Potential prognostic factors for the outcome of DN-aided EMS were also analyzed.

## Materials and methods

### Participant enrollment

**Fig. 1:**
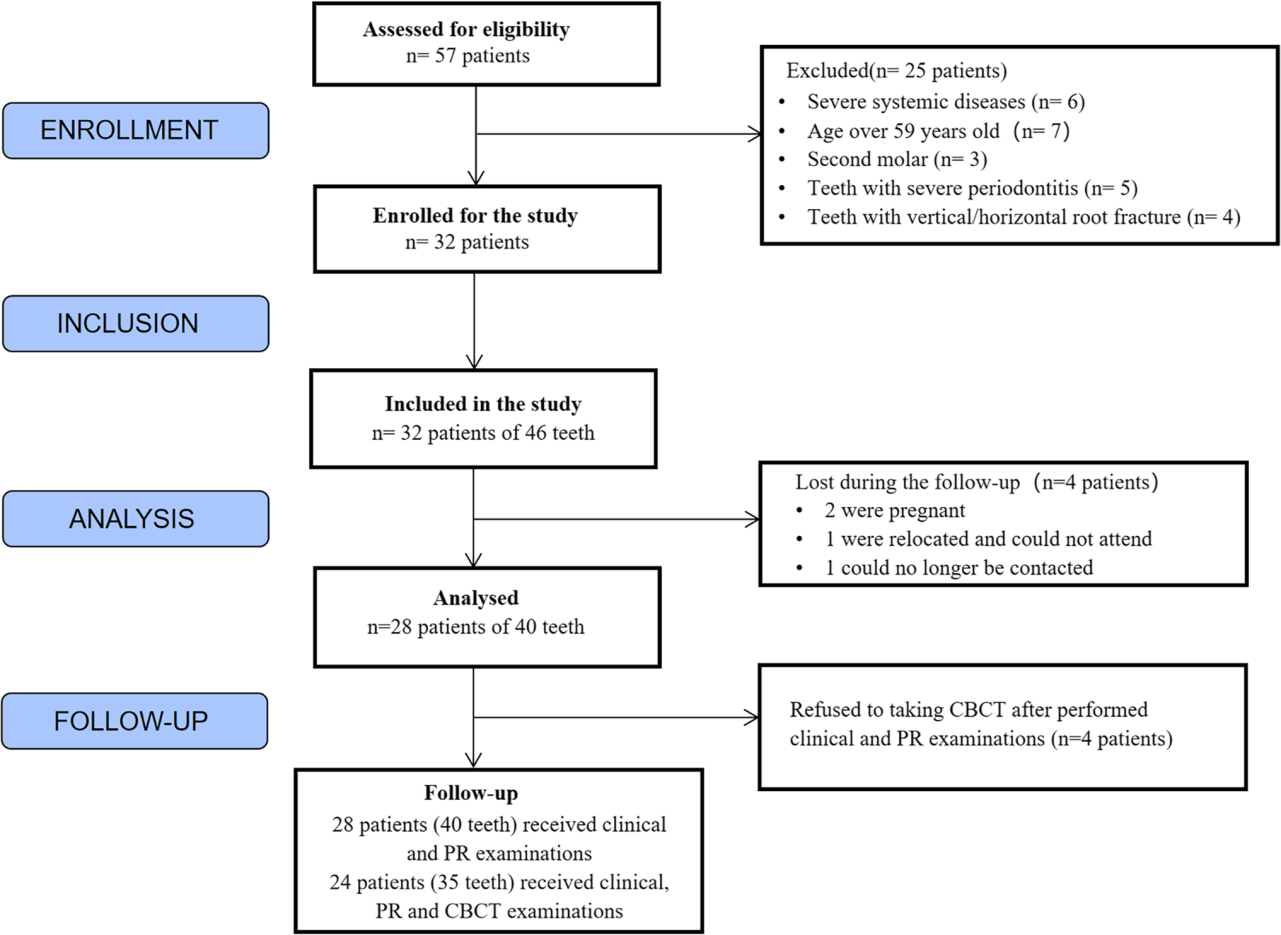
Flowchart showing the flow of participants throughout the study

This study was approved by the Ethics Committee of School and Hospital of Stomatology, Wuhan University (number 2020–06), and registered with the Chinese Clinical Trial Registry (ChiCTR2100042312). Patients who required EMS were enrolled after informed consent was obtained. All patients were informed of the benefits, risks, and required follow-up assessments. The patients were consecutively treated between November 2020 and January 2022. Follow-up assessments were performed at least 1 year postoperatively. The flow of participants throughout the study is shown in Fig. 1. The inclusion and exclusion criteria were as follows.

Inclusion criteria:

- Diagnosis of symptomatic/asymptomatic apical periodontitis in previously root canal treated teeth
- Patients aged 18–59 years

Exclusion criteria:

- Severe systemic diseases (diabetes, osteoporosis, heart disease, etc.)
- Pregnant and lactating women
- Second or third molars
- Teeth with severe periodontitis
- Teeth with vertical/horizontal root fracture, perforated root canals, or root resorption

### DN-aided EMS workflow

**Fig. 2:**
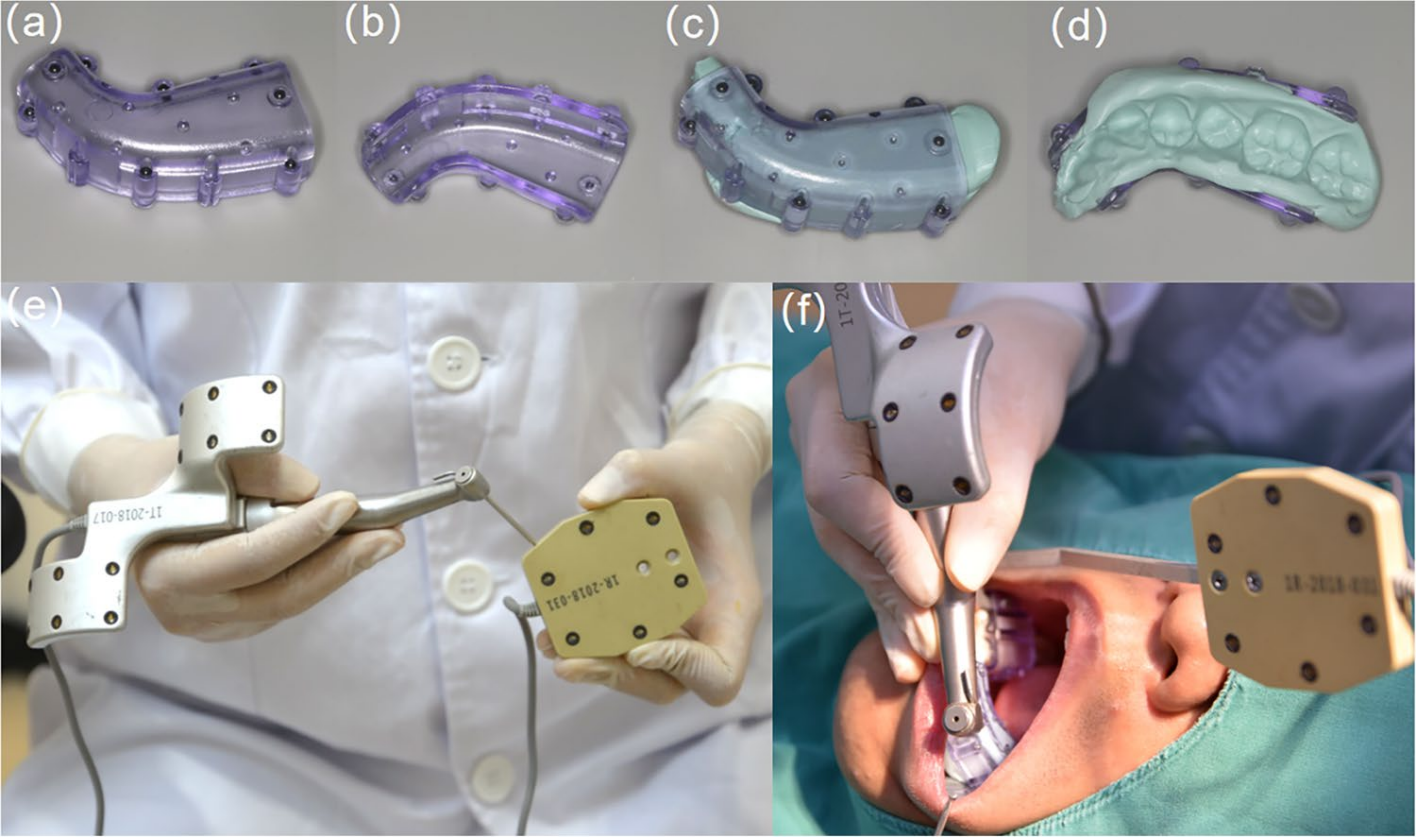
Workflow of dynamic navigation-aided-endodontic microsurgery preoperatively. **a**, **b** Thermoplastic device. **c**, **d** Thermoplastic device filled with silicone impression material. **e** Calibration. **f** Registration

The DN-aided EMS was performed by an experienced endodontist with 8 years of EMS experience and had performed 20-sample guided osteotomy and root-end resection in vitro [21]. The procedures and principles of the EMS were followed the guidelines proposed by Kim and Kratchman [2]. Preoperative PA was performed using an X-ray machine (Planmeca Intra, Helsinki, Finland), exposing the sensors at 60 kVp, 7.0 mA, and exposure time adjusted for the location of the surgical teeth in the dental arch. Preoperative CBCT (Morita, J. Morita, MFG. CORP, Kyoto, Japan) imaging was performed with a thermoplastic device placed on the target tooth of the patient, which embeds nine or 10 radiopaque fiducial markers (Fig. 2a–d). CBCT scanning used the following parameters: tube voltage, 90 kVp; tube current, 7.0 mA; field of view, 80 × 80 mm; and voxel size, 0.16 mm. The DICOM dataset of CBCT was imported into DN software (DHC-ENDO1, DCARER Medical Technology, Suzhou, China) to establish navigation path planning. The drilling entry point, end point, and angle were virtually planned. Calibration and registration process was performed before the osteotomy. Calibration was conducted to match the handpiece-tracking array and the reference device (Fig. 2e). The reference device was fixed to the non-operative area of the dentition (Fig. 2f). Marker-based registration was then performed by completing the matching between the thermoplastic device with 6 radiopaque fiducial markers and the responding area in the 3D reconstruction of the patient’s dentition (Fig. 2f). Full mucoperiosteal flap elevation was used in all cases. Under DN guidance (Fig. 4m), a trephine bur with an outer diameter of 4 mm and a working length of 15.1 mm trephine (818–102, Changsha Tiantian Dental Equipment Co., LTD, Changsha, China) was used to perform the osteotomy and 3 mm of root-end resection. The remaining clinical procedures, including periapical curettage, methylene blue staining, root-end retrograde preparation, retrograde filling with iRoot BP Plus (Innovative Bioceramics, Vancouver, BC, Canada), and sutures, were performed using an endodontic microscope (OPMI PICO; Carl Zeiss, Göttingen, Germany).

### Follow-up and outcome assessment

All patients were telephonically requested to attend the follow-up appointments at least 1-year postoperatively. The reason for this loss was recorded if the patient was unable to attend the follow-up. Clinical success was defined as the absence of any signs and/or symptoms, including pain or swelling, tenderness to percussion or palpation, mobility, sinus tract formation, or periodontal pocket. The parameters of PA and CBCT at follow-up were consistent with preoperative values. Paralleling technique was used to achieve the same angle between the sensor and the teeth before and after the EMS. The radiographic evaluations were performed as follows:

- Two-dimensional (2D) radiographic healing assessment

The 2D radiographic outcomes of periapical films were defined by Rud et al. (1972) and Molven et al. (1987) [6, 7] and classified into four categories: complete healing, incomplete healing, uncertain healing, and unsatisfactory healing (Table 1). Complete healing and incomplete healing were dichotomized as successful radiographic evaluation. Uncertain healing and unsatisfactory healing were dichotomized as failures in the radiographic evaluation.

- 3D radiographic healing assessment

**Table 1:** Two-dimensional (2D) and three-dimensional (3D) radiographic healing assessment of the endodontic microsurgery

	2D radiographic healing assessment criteria	3D radiographic healing assessment criteria
Complete healing	(A) Re-formation of periodontal space of normal width and lamina dura to be followed around the apex (B) Slight increase in width of apical periodontal space, but less than twice the width of non-involved parts of the root (C) Tiny defect in the lamina dura adjacent to the root filling (D) Complete bone repair; bone bordering the apical area does not have the same density as surrounding non-involved bone (E) Complete bone repair; no apical periodontal space can be discerned	(A) Re-formation of periodontal space of normal width and lamina dura over the entire resected and un-resected root surfaces (B) Slight increase in width of apical periodontal space over the resected root surface, but less than twice the width of non-involved parts of the root (C) Small defect in the lamina dura surrounding the root-end filling (D) Complete bone repair with discernible lamina dura; bone bordering the apical area does not have the same density as surrounding non-involved bone (E) Complete bone repair. Hard tissue covering the resected root-end surface completely. No apical periodontal space can be discerned
Incomplete/limited healing	Rarefaction has decreased in size or remained stationary and is characterized by one or more of the following findings: (A) Bone structures are recognized within the rarefaction (B) The periphery of the rarefaction is irregular and may be demarcated by a compact bone border (C) The rarefaction is located asymmetrically around the apex (D) The connection of the rarefaction with the periodontal space is angular	Complete healing can be observed in immediate vicinity of the resected root surface, but the site demonstrates one of the following conditions: (A) The continuity of the cortical plate is interrupted by an area of lower density (B) A low-density area remains asymmetrically located around the apex or has an angular connection with the periodontal space (C) Bone has not fully formed in the area of the former access osteotomy (D) In areas with preexisting periodontal disease or physiologic fenestrations, un-resected root surfaces do not demonstrate bone coverage and/or periodontal reattachment
Uncertain healing	The rarefaction has decreased in size and with one or more of the following characteristics: (A) The radiolucency is larger than twice the width of the periodontal space (B) The radiolucency is bordered by lamina-dura like bone structures (C) The radiolucency has a circular or semicircular periphery (D) The radiolucency is located symmetrically around the apex as a funnel-shaped extension of the periodontal spate (E) Bony structures are discernible within the bony cavity	The volume of the low-density area appears decreased and demonstrates one of the following conditions: (A) The thickness is larger than twice the width of the periodontal space (B) The location is symmetrically around the apex as a funnel-shaped extension of the periodontal space
Unsatisfactory healing	The rarefaction has enlarged of is unchanged	The volume of the low-density area appears enlarged or unchanged

The assessments were based on Rud’s and Molven’s criteria^1, 2^ for 2D healing and modified Penn criteria^3^ for 3D healing, respectively

The 3D radiographic outcomes of CBCT imaging were defined using modified PENN 3D criteria [12] and classified into four categories: complete healing, limited healing, uncertain healing, and unsatisfactory healing (Table 1). Complete healing and limited healing were dichotomized as successful radiographic evaluation. Uncertain healing and unsatisfactory healing were dichotomized as failures in the radiographic evaluation.

### Outcome designations

All radiographs were assessed independently by two examiners (Observer A, C.C. and Observer B, W.Z.) and the Cohen kappa value for agreement was calculated. Any disagreement regarding the 2D or 3D readings was resolved by discussion until a consensus was reached. Radiographic and clinical data were combined to determine the final outcomes, as follows:

- Success, clinical success, and radiographic outcome success
- Failure, clinical failure, and/or radiographic outcome failure

### Evaluation factors

Evaluation factors possibly influencing prognosis were divided into patient- and tooth-related factors. Patient-related factors included age and sex. Tooth-related factors included tooth position, arch type, maximum lesion size, preoperative lesion volume, the presence/absence of crowns and posts, and root canal filling state, including the quality of lateral seal and length of root filling [22]. The preoperative volume of periapical radiolucency and preoperative maximum diameter in three orientations, including mesiodistal, apicocoronal, and buccolingual of the apical lesions, were determined using Mimics software (Materialize Mimics 21, Leuven, Belgium).

### Statistical analysis

The kappa test was used to calculate the inter-observer and intra-observer reliability amongst the four healing categories. Regarding the intra-observer reliability, the second assessment was carried out by the same examiner 1 month after the initial assessment. For the statistical analysis, the dichotomous outcome of success or failure was used as the dependent variable. The Fisher exact test was used to confirm the presence of significant prognostic factors. All statistical analyses were conducted using SPSS Statistics version 27 (IBM Corp. Released 2020, Armonk, NY, USA). A value of *P* < 0.05 was considered statistically significant.

## Results

In the 2D analysis, the intra-observer agreement results were substantial agreement (*K* = 0.73) for Observer A and substantial agreement (*K* = 0.74) for Observer B. In the 3D analysis, the intra-observer agreement results were perfect agreement (*K* = 0.95) for Observer A and perfect agreement (*K* = 0.89) for Observer B. Regarding the inter-observer reliability, the 2D and 3D analysis showed a substantial agreement (*K* = 0.79) and perfect agreement (*K* = 0.95), respectively.

After assessment and enrollment, a total of 46 treated teeth in 32 patients (14 men and 18 women) received DN-aided EMS and included in the study. Forty teeth from 28 patients were available for follow-up. The recall rate was 87.5% (28/32) for patients and 87% (40/46) for teeth. Four patients dropped out for the following reasons: two patients were pregnant, one relocated to another city and could not participate in the follow-up, and one could no longer be contacted.

Of the 28 patients, 13 were men and 15 were women with a mean age of 31.4 years. Of the 40 teeth, 32 were in the maxilla and 8 were in the mandible, including 29 anterior and 11 posterior teeth. The follow-up occurred between 12 and 20 months after surgery. The mean follow-up duration was 13 months. Four patients with five teeth who underwent clinical examination and PA refused CBCT. Finally, 28 patients with 40 teeth underwent clinical and PA examinations, and 24 with 35 teeth underwent clinical, PA, and CBCT examinations (Fig. 1).

**Fig. 3:**
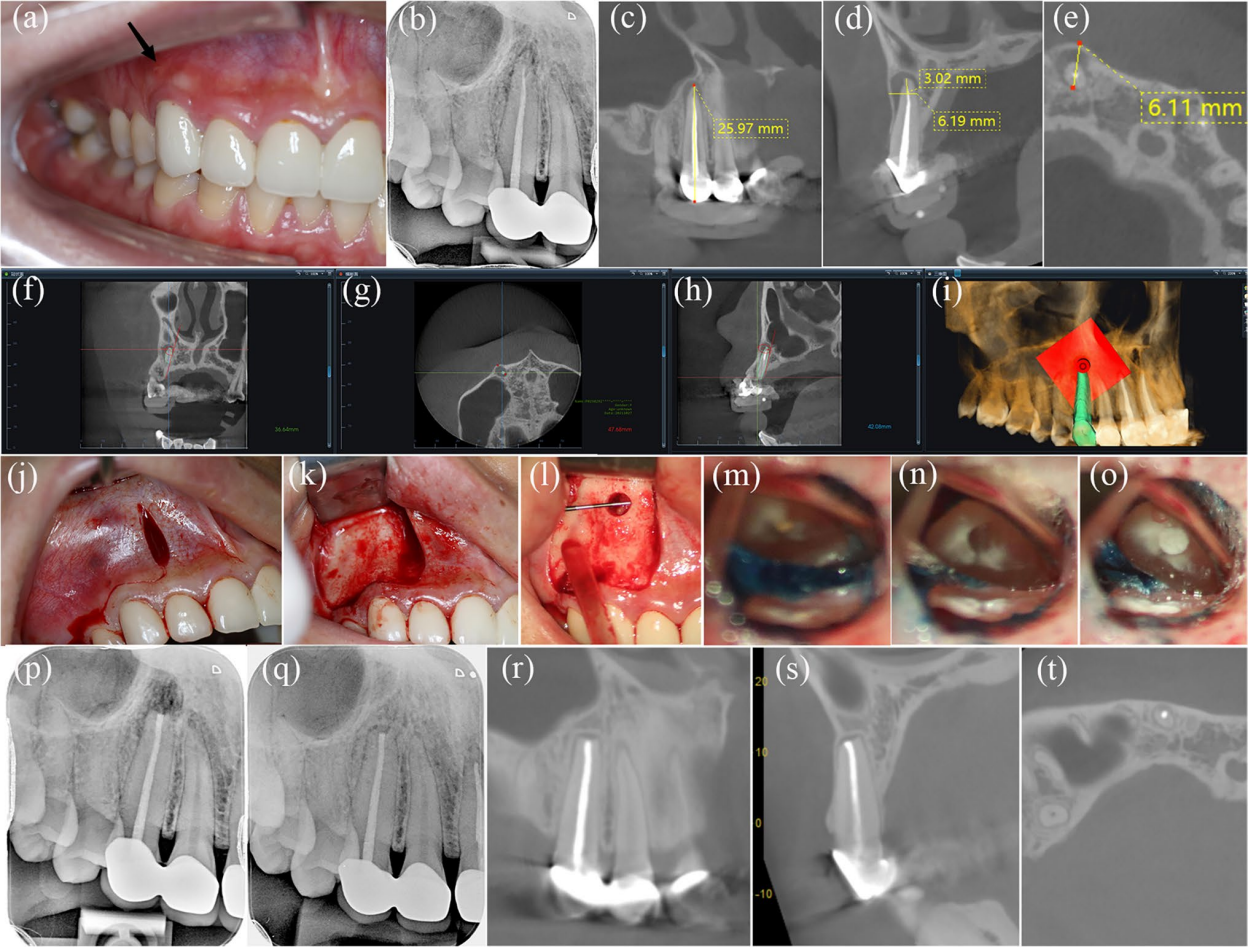
Typical anterior dynamic navigation (DN)–aided endodontic microsurgery case in tooth 13. **a** Preoperative photograph; the black arrow indicates the sinus tract. **b** Preoperative radiograph. **c**–**e** Preoperative coronal, sagittal, and axial cone-beam computed tomography (CBCT) sections and measurement. **f**–**h** Surgical path planning in DN software of coronal, axial, and sagittal view, respectively. **i** Three-dimensional view of surgical path planning in DN software. **j** Incision. **k** Flap elevation. **l** Diameter of osteotomy. **m** Methylene blue staining. **n** Retrograde preparation. **o** Retrograde filling. **p** Immediate postoperative radiograph. **q** One-year follow-up radiograph. **r**–**t** One-year follow-up CBCT of coronal, sagittal, and axial view

**Fig. 4:**
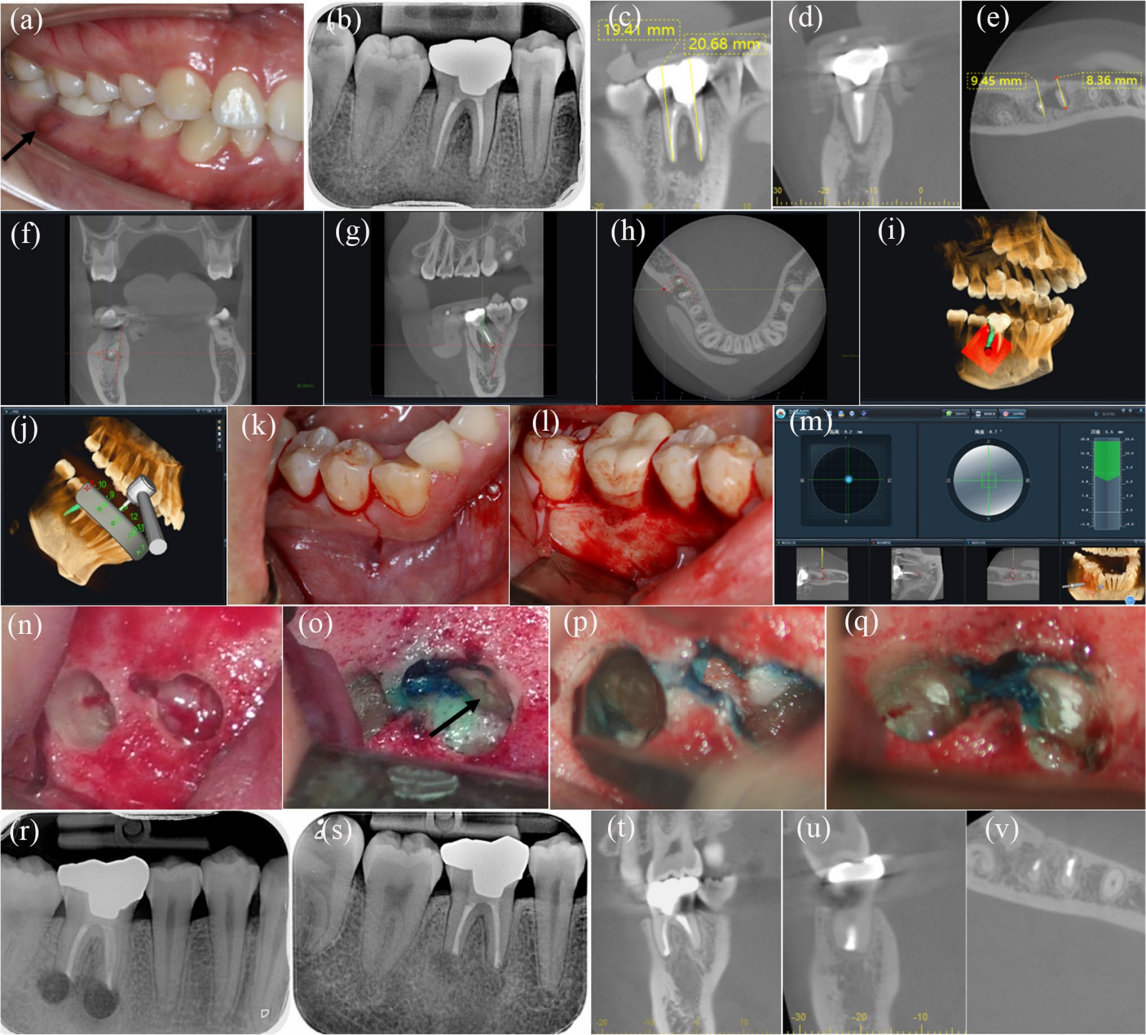
Typical posterior dynamic navigation (DN)–aided endodontic microsurgery case in tooth 46. **a** Preoperative photograph; the black arrow indicates the sinus tract. **b** Preoperative radiograph. **c**–**e** Preoperative sagittal, coronal, and axial cone-beam computed tomography (CBCT) sections and measurement. **f**–**h** Surgical path planning in DN software of coronal, axial, and sagittal view, respectively. **i** Three-dimensional view of surgical path planning in DN software. **j** Registration. **k** Incision. **l** Flap elevation. **m** Real-time views in the screen of DN system. **n** After guided osteotomies and root-end resections. **o**, **p** Retrograde preparation of mesial and distal root, respectively; black arrows indicate the root resection plane. **q** Retrograde filling. **r** Immediate postoperative radiograph. **s** One-year follow-up radiograph. **t**–**v** One-year follow-up CBCT of sagittal, coronal, and axial view

At the follow-up appointment, two teeth of two patients exhibited signs of a sinus tract and unsatisfactory healing on radiological examination. No signs or symptoms were observed in the remaining teeth. The characteristics of the included DN-aided EMS cases with follow-up time are demonstrated in Table 2. The healing outcomes of PA and CBCT are summarized in Table 3. Combined clinical and radiographic data demonstrated that the success rates of DN-aided EMS were 95% (38/40) and 94.3% (33/35), as assessed using PA and CBCT, respectively. Representative anterior and posterior cases are shown in Figs. 3 and 4, respectively. The distribution and analysis of the cases per variable category are summarized in Table 4. No factor listed in Table 4 was associated with significant differences.

**Table 2:** The characteristics of the included dynamic navigation-aided-endodontic microsurgery cases with follow-up time

Tooth number	Roots treated	Age	Sex	Follow-up time(months)	Outcome of clinical evaluation	Outcome designation of PA	Outcome designation of CBCT
11	Single root	28	F	12	Asymptomatic	Complete healing	Complete healing
21	Single root	28	F	12	Asymptomatic	Complete healing	Complete healing
22	Single root	28	F	12	Asymptomatic	Incomplete healing	Limited healing
21	Single root	18	M	13	Asymptomatic	Complete healing	Complete healing
22	Single root	18	M	13	Asymptomatic	Complete healing	Complete healing
23	Single root	18	M	13	Asymptomatic	Complete healing	Complete healing
21	Single root	19	F	12	Asymptomatic	Incomplete healing	Limited healing
21	Single root	40	F	12	Asymptomatic	Complete healing	Limited healing
22	Single root	40	F	12	Asymptomatic	Complete healing	Limited healing
13	Single root	41	F	17	Asymptomatic	Incomplete healing	\
14	Single root	41	F	17	Asymptomatic	Incomplete healing	\
12	Single root	22	F	20	Asymptomatic	Complete healing	Complete healing
11	Single root	22	F	20	Asymptomatic	Complete healing	Complete healing
21	Single root	22	F	20	Asymptomatic	Complete healing	Complete healing
21	Single root	29	F	12	Asymptomatic	Complete healing	Complete healing
12	Single root	34	F	12	Asymptomatic	Complete healing	Limited healing
11	Single root	29	M	19	Asymptomatic	Incomplete healing	Uncertain healing
12	Single root	29	M	19	Asymptomatic	Complete healing	Complete healing
12	Single root	37	F	13	Asymptomatic	Complete healing	Limited healing
13	Single root	37	F	13	Asymptomatic	Incomplete healing	Limited healing
11	Single root	49	F	12	Asymptomatic	Complete healing	Complete healing
41	Single root	26	F	12	Asymptomatic	Complete healing	Complete healing
11	Single root	29	M	12	Asymptomatic	Complete healing	Limited healing
21	Single root	29	M	12	Asymptomatic	Incomplete healing	Uncertain healing
22	Single root	29	M	12	Asymptomatic	Incomplete healing	Limited healing
11	Single root	34	F	12	Sinus tract	Unsatisfactory healing	\
36	M, D	27	F	12	Asymptomatic	Incomplete healing	Limited healing
36	M, D	26	M	12	Asymptomatic	Complete healing	\
36	M, D	18	M	12	Asymptomatic	Complete healing	Complete healing
34	Single root	42	M	12	Asymptomatic	Complete healing	Complete healing
22	Single root	38	M	12	Asymptomatic	Incomplete healing	Limited healing
21	Single root	59	M	14	Asymptomatic	Incomplete healing	Limited healing
11	Single root	35	M	12	Asymptomatic	Complete healing	Limited healing
46	M, D	22	M	12	Asymptomatic	Complete healing	Complete healing
36	M, D	35	M	12	Asymptomatic	Incomplete healing	Limited healing
46	M, D	18	F	13	Asymptomatic	Complete healing	Limited healing
16	MB, DB, P	27	M	12	Asymptomatic	Complete healing	Complete healing
13	Single root	26	F	12	Asymptomatic	Complete healing	Complete healing
26	MB, DB, P	24	M	12	Sinus tract	Unsatisfactory healing	\
16	MB, DB	48	F	12	Asymptomatic	Complete healing	Limited healing

*M* mesial, *D* distal, *MB* mesial buccal, *DB* distal buccal, *P* palatal, *F* female, *M* male, *PA* periapical radiography, *CBCT* cone-beam computed tomography; \, tooth did not undergo CBCT examination

**Table 3:** A summary of results from two-dimensional and three-dimensional radiographic healing assessment of 1 year after the dynamic navigation-aided endodontic microsurgery

	2D PA (*n* %)	3D CBCT (*n* %)
*N* (teeth)	40	35
Complete healing	26 (65%)	17 (48.6%)
Incomplete/limited healing	12 (30%)	16 (45.7%)
Uncertain healing	0	2 (5.7%)
Unsatisfactory healing	2 (5%)	0
Success rate (%)	95%	94.3%

*PA* periapical radiography, *CBCT* cone-beam computed tomography

**Table 4:** Distribution and analysis of cases per variable/category

Factors	PA (*N* = 40)			CBCT (*N* = 35)			*P* value	
	*N*(teeth)	Success	Failure	*N*(teeth)	Success	Failure	PA	CBCT
Sex							> 0.99	0.202
Female	22	21	1	19	19	0		
Male	18	17	1	16	14	2		
Age							> 0.99	0.536
≤ 30 y	25	24	1	23	21	2		
> 30 y	15	14	1	12	12	0		
Tooth type							0.479	> 0.99
Anterior	29	28	1	27	25	2		
Posterior	11	10	1	8	8	0		
Arch type							> 0.99	> 0.99
Maxilla	32	30	2	28	26	2		
Mandible	8	8	0	7	7	0		
Preoperative volume (*V* (mm^3^))							> 0.99	> 0.99
≤ 300 mm^3^	27	26	1	23	22	1		
> 300 mm^3^	13	12	1	12	11	1		
Maximum lesion size							> 0.99	> 0.99
≤ 10 mm	20	19	1	16	15	1		
> 10 mm	20	19	1	19	18	1		
Crown							> 0.99	0.519
Absent	15	14	1	13	13	0		
Present	25	24	1	22	20	2		
Post							> 0.99	0.496
Absent	28	27	1	25	24	1		
Present	12	11	1	10	9	1		
Root canal filling							0.492	0.229
Inadequate	18	18	0	18	18	0		
Adequate	22	20	2	17	15	2		

*PA* periapical radiography, *CBCT* cone-beam computed tomography

The adequacy of root canal filling means both the adequate lateral seal and length

## Discussion

This prospective study was aimed at evaluating the 1-year clinical and radiological outcomes and potential prognostic factors of novel navigation-guided EMS. The concept of guided endodontic surgery, including SN and DN technology, overcame the challenges of free-hand EMS up to an extent and allowed for a more appropriate length and bevel for root-end resection. Of all the published in vivo SN and DN endodontic surgery studies, except for one retrospective study, all were case report/series with favorable clinical and radiological outcomes (Table 5). To our knowledge, this is the first prospective study to investigate the prognosis of DN-aided EMS. As healing mainly happened within 1 year postoperatively, a 1-year follow-up is considered a feasible indicator of the final prognosis outcome [23]. In this study, a mean follow-up period of 13 months was deemed adequate for the analysis of the clinical and radiographic assessments. The success rates of DN-aided EMS 1 year postoperatively in PA and CBCT were both more than 94.3%, which is similar to the freehand EMS study [1].

**Table 5:** In vivo studies of the prognosis of guided endodontic microsurgery

Guided methods	Author(year)	Article type	Sample size/case number	DN system /SN printer	Bur			Follow-up time (months)	Evaluation method	Radiological outcome	Success rate (%)
					Type	Diameter (mm)	Speed (rpm)				
DN	Chen et al. (2022, Ours) [21]	Original articles	28 patients (40 teeth)	DHC-ENDO1 (DCARER Medical Technology)	Trephine (818–102, Changsha Tiantian Dental Equipment Co., Ltd, China)	4	1200	12–20	PA and CBCT	Showed in Table 3	PA: 95% CBCT: 94.3%
	Lu et al. (2022) [38]	Case report	1	X-guide (X-Nav Technologies)	\	5.5	\	5	PA	Significant bone healing	\
	Fu et al. (2022) [39]	Case reports	3	DHC-ENDO1 (DCARER Medical Technology)	Trephine (818–102, Changsha Tiantian Dental Equipment Co., Ltd, China)	4	1200	3–9	PA	Increased bone density	\
	Gambarini et al. (2019) [40]	Case report	1	Navident (ClaroNav)	Round diamond bur (#801-018C, SS White, USA)	3	\	6	PA	Complete healing	\
SN	Chaves et al. (2022) [41]	Case report	1	MoonRay S100 (SprintRay Inc.)	Ultrasonic surgical tips (Piezotome; Acteon, France)	\	\	12	CBCT	Bone healing	\
	Buniag et al. (2021) [20]	Original articles	23 patients (24 teeth)	Objet260 Connex3 (Stratasys Ltd.)	\	5 or less	900–1000	12–28	PA	Complete healing, 20 Reductive healing, 2 Failures, 2	91.7%
	Benjamin et al. (2020) [42]	Case series	3	Formlabs 2 (Formlabs Inc.)	Trephine (Zimmer Biomet 3i, USA)	4	1000	\	\	\	\
	Kim et al. (2020) [43]	Case report	1	Object EDEN260V (Stratasys Ltd.)	Piezoelectric saws	\	\	12	PA	Signs of healing	\
	Popowicz et al. (2019) [44]	Case reports	2	Prusa i3 MK2S (Prusa Research s.r.o)	Trephine (No227A.204.040; Komet, Germany)	3	1000	7–8	CBCT	Complete 3D healing	\
	Giacomino et al. (2018) [45]	Case reports	3	Objet260 Connex3 (Stratasys Ltd.)	Trephine (Biomet 3i, LLC, USA)	5 or 6	1200	1–3	\	\	\
	Antal et al. (2018) [46]	Case series	11 patients (14 roots)	ProJet MD 3510 (3D system Corporation)	Trephine (Hager & Meisinger, Germany)	4.21	\	6	\	\	\
	Ye et al. (2018) [47]	Case report	1	3510SD (3D system Corporation)	Trephine (Meisinger, Germany)	4	\	12	PA	No periapical radiolucency	\
	Ahn et al. (2018) [48]	Case report	1	Object EDEN260V (Stratasys Ltd.)	Anchor drill	1.5	\	\	\	\	\
	Strbac et al. (2017) [49]	Case report	1	Objet350 Connex 3 (Stratasys Ltd.)	Piezoelectric saw (Piezomed, W&H Dentalwerk GmbH, Austria)	\	\	12	CBCT	Complete healing	\
	Liu et al. (2014) [50]	Case report	1	Objet Connex 500 (Stratasys Ltd.)	\	\	\	\	\	\	\

*DN* dynamic navigation, *SN* static navigation, *PA* periapical radiography, *CBCT* cone-beam computed tomography; \, not mentioned

Although histology is considered the gold standard for the evaluation of periapical healing, it is ethically forbidden to obtain tissue cores from patients at follow-up. Previous animal studies have shown that CBCT provides a similar result to histology in healing outcome [24, 25]. In addition, some studies reported 27–33% disagreement on the diagnosis between PA and CBCT in the follow-up images [8, 26, 27], as CBCT presents lesion changes in the cortical plate and trabecular bone more accurately. The present study also demonstrated differences in the outcomes between these two radiographic methods. In the present study, eight teeth taken for complete healing based on the 2D classification fell into the category of limited healing patterns in the PENN 3D criteria, and two teeth taken for incomplete healing based on the 2D classification fell into the category of uncertain healing in the PENN 3D criteria. CBCT demonstrated more radiographic defects in 3D views and resulted in a lower healing rate than PA [28]. These defects are not always associated with clinical failure but might indicate incomplete or defective healing in radiological outcomes [29]. A longer follow-up period is needed for a more reliable assessment of these controversial radiological outcomes.

At the 1-year follow-up, two patients exhibited both clinical and radiological failures. Failure occurred in tooth 11 in a 34-year-old woman and tooth 26 in a 24-year-old man (Table 2). After root-end resection and methylene blue staining, these two teeth were found to be suffered from apical dentin microfractures, one of which occurred in the palatal side of the resected root and the other occurred in the mesial side of the resected palatal root. Otterson et al. (2020) illustrated that, compared to freehand EMS, targeted EMS with trephine does not increase the risk of dentinal microcracks [30]. Some studies have illustrated that the development of dentinal microfractures in endodontically treated teeth is directly associated with shaping, cleaning, and filling procedures [31–33]. Moreover, Arias et al. (2021) reported that mastication and other functions of the patient are important factors in the presence of cracks [34]. Although the cracked dentine was eliminated by further resection of the root during surgery, microcracks may exist and can be further prolong. In addition, the factors of occlusal force and mastication performance of the patient still existed and might have led to the progressive development of microcracks, compromising healing. In the present study, four patients refused to undergo CBCT after receiving clinical and PA examinations. Of these four patients, two with two teeth were diagnosed with unsatisfactory healing by PA, as previously described. Therefore, we reasonably speculate that the actual CBCT success rate was lower than the current rate of 94.3%, which is a limitation of this study.

Previous freehand EMS studies have reported that tooth and arch types are important factors influencing success rates [15–18]. The low success rate of posterior and mandibular teeth may result from anatomical obstacles in visualization and access during osteotomy and root-end resection. In addition, altered sensation, associated with the injury of mental and inferior alveolar nerves, may happen after EMS procedures. The long-term or permanent altered sensation can have a serious impact on patient’s quality of life and even attribute to medicolegal consequences. A previous study reported a 14% incidence of altered sensation after mandibular premolar-molar surgery [35]. In the present study, six mandibular molar cases and one mandibular premolar case were included and no intraoperative complications occurred. Technically challenging cases, including the palatal root of the maxillary first molar, maxillary molar root adjacent to the maxillary sinus, the curve distal root of the mandibular first molar, maxillary and mandibular premolars, and root tip in approximation to the mental nerve, were included in this study (Table 2). However, some of these complexities may have precluded treatment with freehand EMS and are associated with the risk of complications, leading to a decrease in success rate. In our study, no significant differences were found between the arch types (maxilla versus mandible) and tooth types (anterior and posterior). The present study indicates that guided EMS is particularly beneficial in complex cases with thick cortical plates, limited access, and poor visualization.

The advantages of DN-aided EMS can be summarized as follows. First, for the clinician, the main advantage of the use of DN is the possibility of performing the surgical path optimally as designed and avoiding the incidence of iatrogenic errors. Second, excessive osteotomy may occur inadvertently in anatomically complex scenarios of free-hand EMS, particularly in posterior dental arch location. In our study, the diameter of surgical approach of minimal osteotomy with trephine was restricted to 4 mm in all cases. With the development of guided EMS, the conception of patient-centered orientation in minimally invasive operations may be further refined. Third, DN allows the modification of the original surgical path intraoperatively if needed, which is not possible in static guides. Finally, in DN-aided EMS with trephine bur, the cortical plate, cancellous bone, granulomatous tissue, and root tip could be removed simultaneously. Simplification of surgical procedures through DN-aided EMS improves clinical efficiency and may increase the clinical prevalence of EMS overall.

There are certain limitations associated with the DN-aided EMS method. Firstly, the cross-section of the root created by the surgical trephine is not flattened and appears as a curved shape, with the level and bevel of the curved edge determined by the surgical path and diameter of the trephine bur. This can lead to stress concentration on the root-end aspect, which may cause cemental tears over time and affect prognosis, as demonstrated by a finite element analysis investigation [36]. To address this issue, the curved edge is removed using a high-speed handpiece with a diamond or ET18D ultrasonic tip in the present cases. Additionally, the preoperative guided procedures of calibration and registration are complicated and time-consuming. However, this is offset by the reduction in intraoperative time during osteotomy and root-end resection. It is important to note that the DN requires a learning curve to develop a stable and consistent workflow and reduce operator-induced errors. In a previous study, it was recommended that operators practice on 20 or more in vitro samples to eliminate the influence of the learning curve [37]. In the present study, the operator practiced on 20 samples in vitro before operating on patients to achieve proficiency in DN [21]. Finally, the obscuration of the patient’s lips and cheeks, particularly in posterior areas, may prevent the use of trephine to facilitate access in the planned position and angle. Therefore, we proposed the conception of fully guided and half-guided navigation approaches in EMS. In the fully guided approach, DN guides the entire drilling process from the osteotomy to the root-end resection according to the planned position and angle. In contrast, in the half-guided approach, only 1–2-mm osteotomy deep into the bone is performed according to the programming position but not angle, which can assist the clinician in locating the position of the apex. The remaining drilling is then performed by the high-speed handpiece with a diamond.

The lack of a control group is a limitation of this single-cohort study. A further limitation is the relatively small sample size, as this was a single-center study. This may lead to a false-negative result when confirming the significant prognostic factors, and the results should be interpreted with caution. Despite these limitations, the present study provided preliminary and inductive conclusions of the outcome of DN-aided EMS. Further studies with larger sample sizes and randomized controlled trials should be conducted to draw more specific conclusions on the validity and effectiveness of this novel technique.

## Conclusion

In conclusion, the success rate of DN-aided EMS 1 year postoperatively was 95% in 2D healing classifications and 94.3% in 3D healing classifications, which is similar to that of evidence-based freehand EMS studies. Sex, age, tooth type, arch type, preoperative lesion volume, preoperative maximum lesion size, presence/absence of crown, post, and root canal filling state did not affect the success rate of DN-aided EMS. The presented novel DN approach appears to be a predictable and clinically feasible method to assist EMS in guided osteotomy and root-end resection.

## Data Availability

Data to perform these studies are available after a justified request to the authors.
